# Supplementary material for: Unveiling the dynamics of antimicrobial utilization and resistance in a large hospital network over five years: Insights from health record data analysis
Source: PLOS Digit Health. 2023 Dec 29;2(12):e0000424. doi: 10.1371/journal.pdig.0000424 (PMC10756551; doi:10.1371/journal.pdig.0000424)
Supplement: S1 Fig — The prescription patterns A) The prescription of drugs in- and out-patients with the names of antimicrobials exclusive to either group of patients. B) The frequency of top 25 highly prescribed antimicrobials for in- and out-patient groups. (DOCX) [file pdig.0000424.s001.docx]

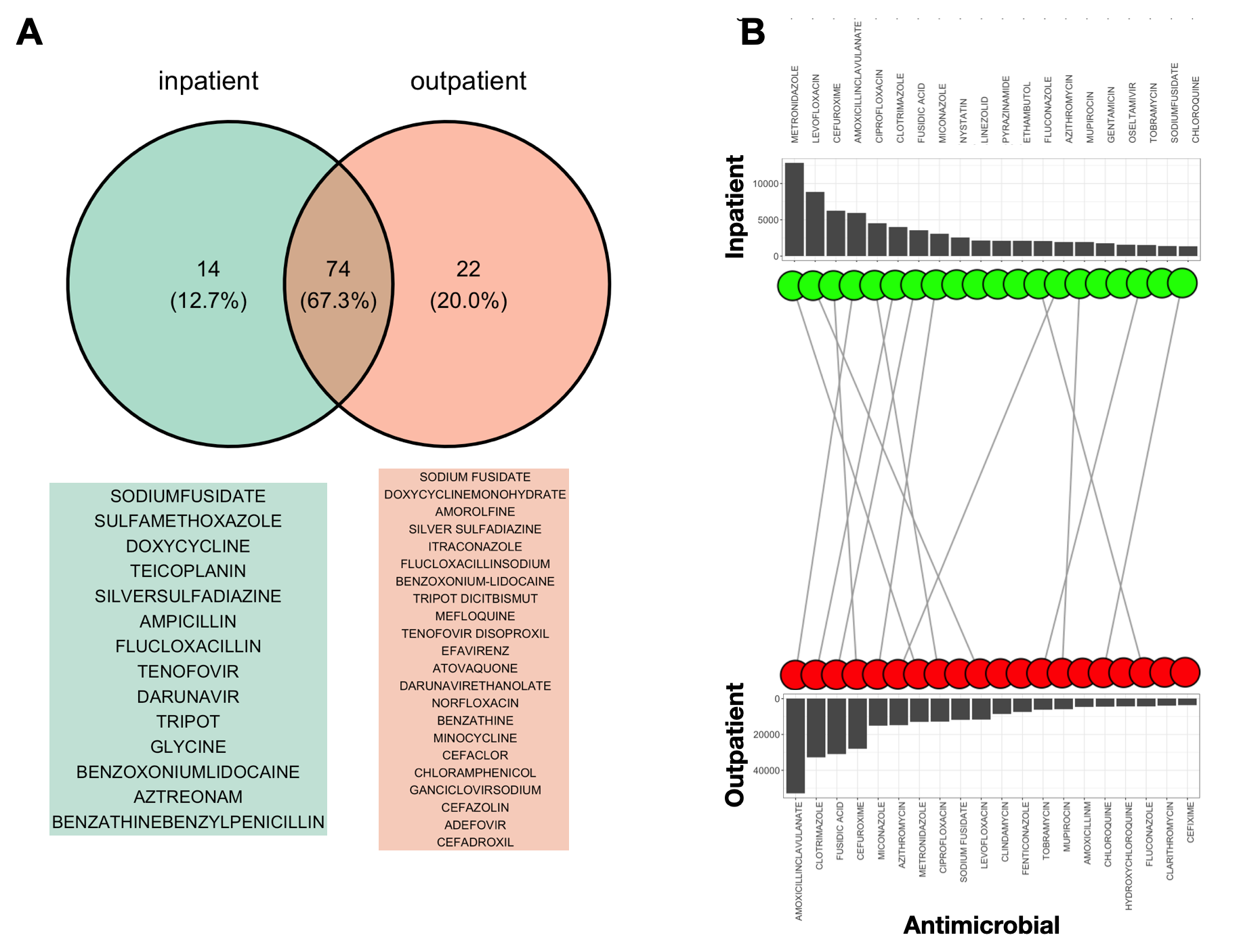


S1 Fig: The prescription patterns A) The prescription of drugs in- and out-patients with the names of antimicrobials exclusive to either group of patients. B) The frequency of top 25 highly prescribed antimicrobials for in- and out-patient groups.
