# Supplementary material for: Unveiling the dynamics of antimicrobial utilization and resistance in a large hospital network over five years: Insights from health record data analysis
Source: PLOS Digit Health. 2023 Dec 29;2(12):e0000424. doi: 10.1371/journal.pdig.0000424 (PMC10756551; doi:10.1371/journal.pdig.0000424)
Supplement: S2 Fig — The link between prescription rate and resistance level and the deaths odds ratio for the A) inpatient and B) outpatient groups. The frequency of prescriptions for the resistant and susceptible strains for the prescription of the same drugs across the organisms. The upper panel shows the odds-ratio for resistance within the patients with the prescription for the antimicrobials. (DOCX) [file pdig.0000424.s002.docx]

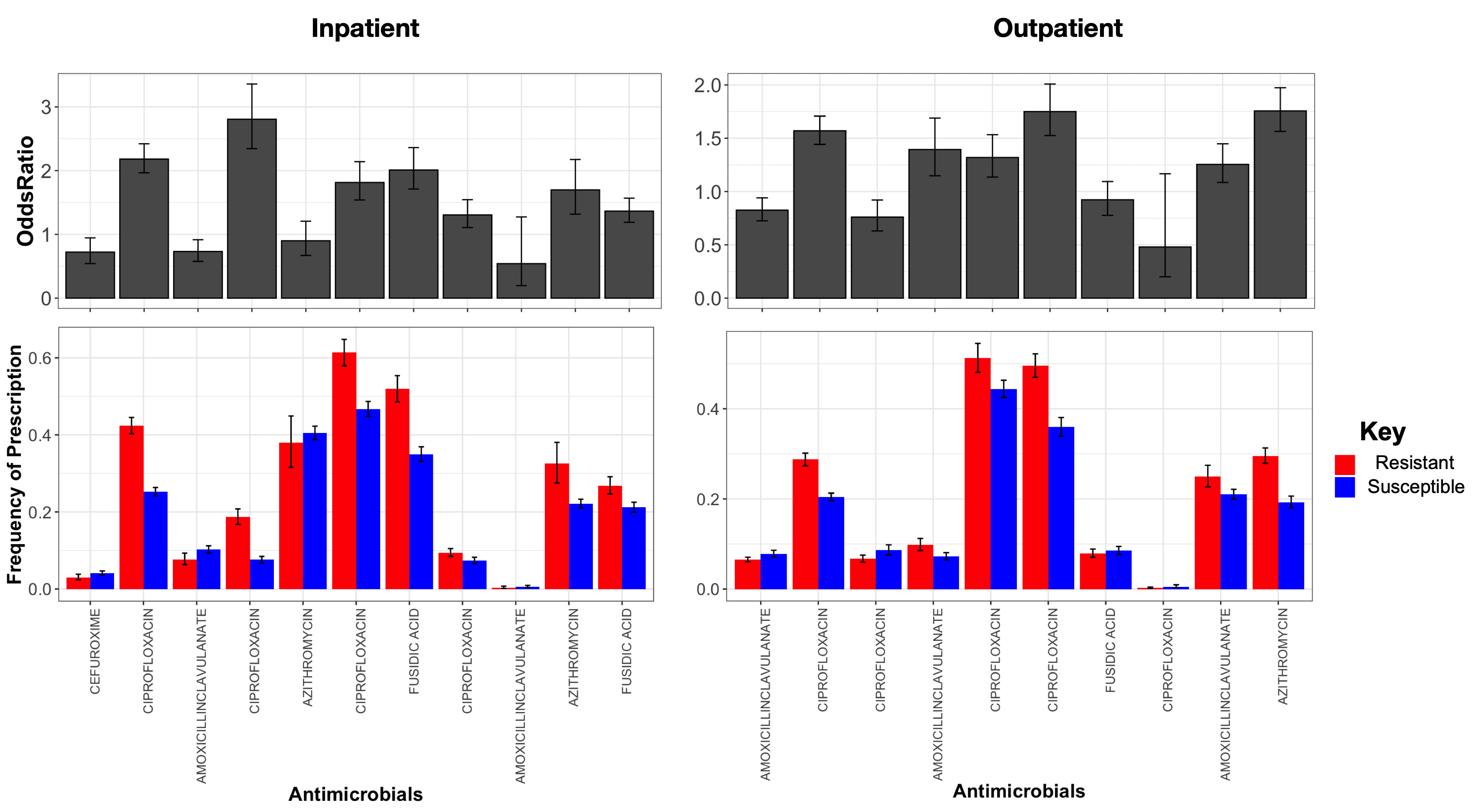


S2 Fig: The link between prescription rate and resistance level and the deaths odds ratio for the A) inpatient and B) outpatient groups. The frequency of prescriptions for the resistant and susceptible strains for the prescription of the same drugs across the organisms. The upper panel shows the odds-ratio for resistance within the patients with the prescription for the antimicrobials.
