# Supplementary material for: Unveiling the dynamics of antimicrobial utilization and resistance in a large hospital network over five years: Insights from health record data analysis
Source: PLOS Digit Health. 2023 Dec 29;2(12):e0000424. doi: 10.1371/journal.pdig.0000424 (PMC10756551; doi:10.1371/journal.pdig.0000424)
Supplement: S3 Fig — (DOCX) [file pdig.0000424.s003.docx]

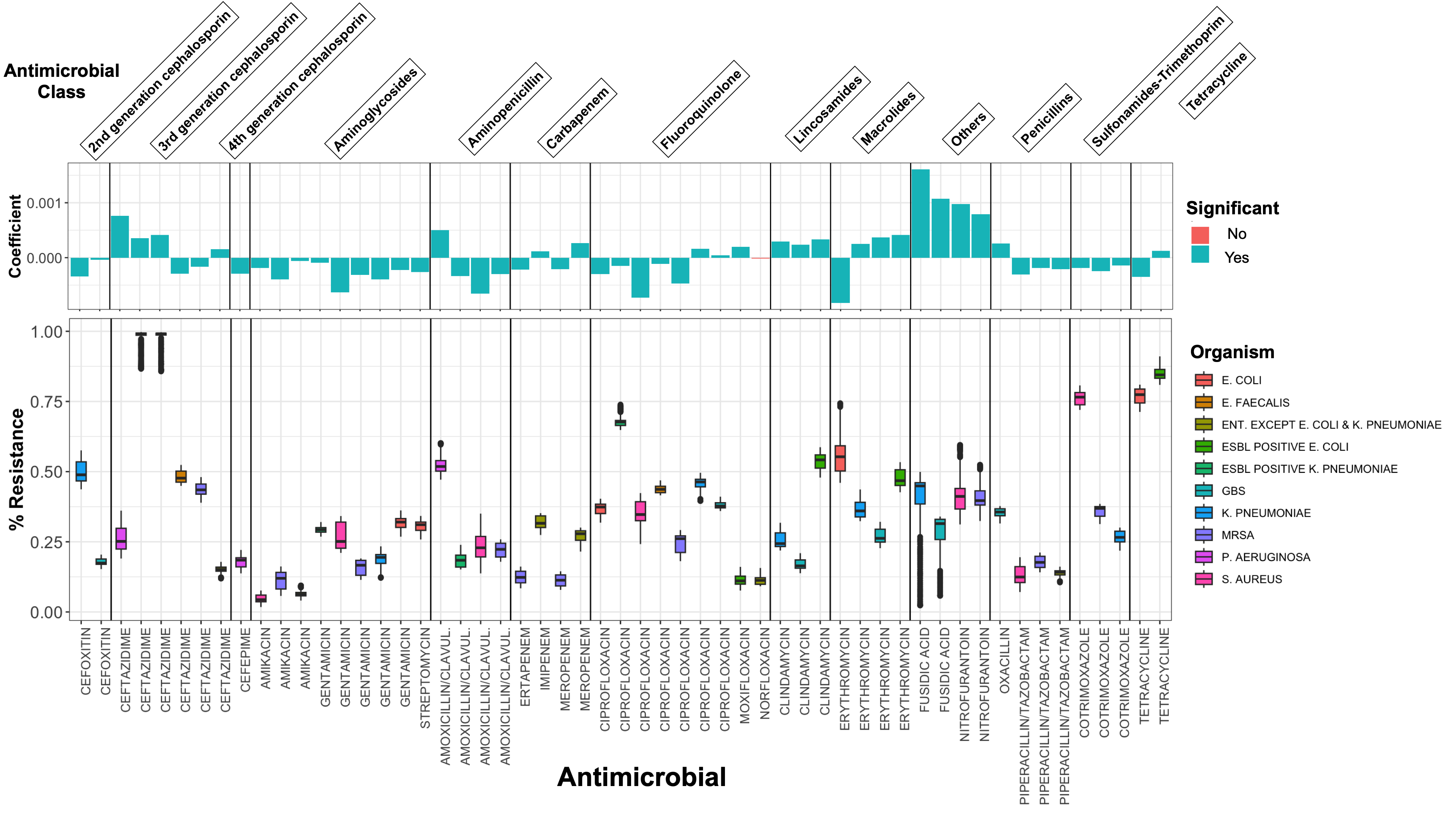


S3 Fig: The distribution of resistance levels for drugs and organisms for which more than 10% difference over time was observed in resistance level. The overall trend for antimicrobials obtained from start and end points in 2022 and 2017.
