# Supplementary material for: Unveiling the dynamics of antimicrobial utilization and resistance in a large hospital network over five years: Insights from health record data analysis
Source: PLOS Digit Health. 2023 Dec 29;2(12):e0000424. doi: 10.1371/journal.pdig.0000424 (PMC10756551; doi:10.1371/journal.pdig.0000424)
Supplement: S4 Fig — The blue line shows the onset of COVID-19. (DOCX) [file pdig.0000424.s004.docx]

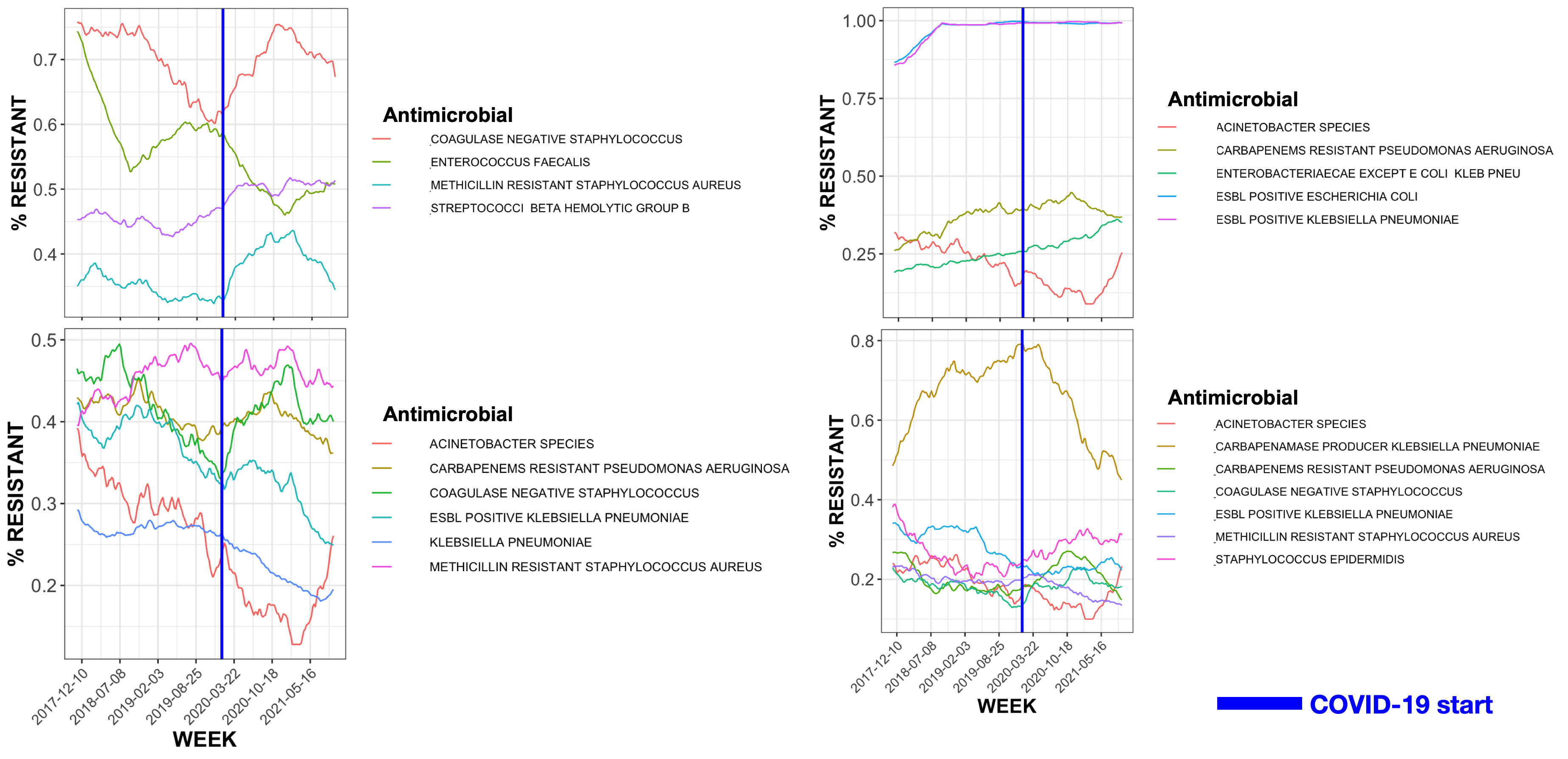


S4 Fig: Resistance trends for the same drugs across different organisms. The blue line shows the onset of COVID-19.
