# Supplementary material for: Unveiling the dynamics of antimicrobial utilization and resistance in a large hospital network over five years: Insights from health record data analysis
Source: PLOS Digit Health. 2023 Dec 29;2(12):e0000424. doi: 10.1371/journal.pdig.0000424 (PMC10756551; doi:10.1371/journal.pdig.0000424)
Supplement: Attachment — Submitted filename: ResponseLetter_submit.docx [file pdig.0000424.s005.docx]

Dear Prof. Wang,

We would like to thank you for considering our manuscript for publication in *PLoS Digital Health.* The reviewer's comments have been invaluable, and we have made a effort to address them comprehensively, as outlined below. We believe that these revisions have substantially enhanced the manuscript's current format, and we are hopeful that you will consider it suitable for publication in *PLoS Digital Health*.

Sincerely yours,

Danesh Moradigaravand and Abiola Senok (on behalf of the other authors)

Additional Editor Comments (if provided):

**See comments from Reviewer 1. Please make your code available before we can accept your paper. Please also at least release part of your raw data for reproducibility. Only sharing post-analysis data is not helpful**.

**Response:** The code is now available in the GitHub repository for the project. The code includes the implementation of statistical methods and visualizations that we used for the figures. We have also included the input datasets for the figures. Since the data includes patient information that could potentially identify them, we are unable to release the raw data in accordance with the regulations of the Dubai Health Authority (DHA) and the Dubai Academic Health Corporation (DAHC). We therefore follow the journals’ guidelines on publishing sensitive data (https://journals.plos.org/digitalhealth/s/data-availability), and now detailed the reason and the contact information for the data access committee for DHA in the data availability statement (Line 280). The data availability statement has therefore changed to the following:

“We provide the code and aggregated data underlying the figures in the GitHub directory of the manuscript ([https://github.com/DaneshMoradigaravand/DubaiAMRProject](https://github.com/DaneshMoradigaravand/DubaiAMRProject)). The raw data includes patients' sensitive information and can be obtained by any user with sufficient justification through communication with the Dubai Scientific Research Ethics Committee at DSREC@dha.gov.ae”

**Reviewers' comments:**

**Reviewer's Responses to Questions**

**Reviewer #1:** Unveiling the dynamics of antimicrobial utilization and resistance in a large hospital network over five years: Insights from health record data analysis 
The aim of this work was to look at impact of COVID-19 on antimicrobial resistance (AMR) evolution as well as ecological trends. It was great to see the open source GitHub but this currently has no code for analysis only some summary csv files. It is fine that the individual patient level data is not available I think as it is healthcare data.

I cannot assess the machine learning analysis, but it seems a reasonable and exciting approach to the free text in the infection diagnosis. I’d also like the authors to check and compare with other methods for looking at the link between resistance and mortality odds: there are some complex statistical methods for looking at competing risks and comorbidities. 

**Response:**

We have now supplied the codes and input data for the figures within the GitHub directory of the project. All population-level data has been made available in the same repository.

Furthermore, we have included the outcomes of competing risks regression survival analysis, specifically through cause-specific hazard modeling, also known as Fine-Gray regression [1, 2]. This statistical technique is utilized to analyze survival data in scenarios involving multiple competing events, affecting the time until the occurrence of a particular event, such as death in our context.

In our study, we specifically examined the impact of patients' infection by resistance strains/strains harboring resistance gene data on the time to death for different drugs and organisms. To achieve this, we employed the 'crr' function for Fine-Gray regression in the 'tidycmprsk' R package. We considered several patient’s attributes, e.g. age, sex, BMI, hospitalization duration and frequency, as well as diagnostic terms such as comorbidities and infection type, as competing causes to death and measured the impact of the presence of resistance strains/genes.

We have now detailed these in the Methods Section (Page 12-14, Lines 234-277) and Results Section (Pages 22-23, Lines 444-473) and updated Figure 6.

**Abstract**

1. Are the 60551 patients those with infection? Or all registered in the network? If former, then should go after the next sentence

**Response:** We examined patients with a confirmed infection, we have now corrected this (Page 2, Line 7)

2. Results: mortality from infection or all mortality?

**Response:** We had data for all mortality (Page 2, Line 19)

3. Results: what prescription practices? New ones?

**Response:** We have now rephrased this to reflect that it is antimicrobial prescription practices (Page 2, Line 17-24).

4. Results: COVI D-19 gap to be removed in last line

**Response:** Corrected (Page 3, Line 24).

5. Conclusions: How do they emphasise the long-term effect of COVID-19? Just two years / technically still in the pandemic?

**Response:** We thank the reviewer for pointing this out. We have therefore rephrased and specially mentioned the period of two years (page3, Lines 25-29).

**Introduction**

1. Line 33: I’d add “in part” as it may also be driven a lot by transmission of resistant pathogens

**Response:** We have added the phrase (Page 4, Line 39)

2. Line 49: Expand EHR first time use

**Response:** This has been done (Page 4, Line 51)

3. Line 55: word missing after “and”

**Response:** “and” has been removed (Page 5, Line 57)

4. Line 68: missing “of”

**Response:** We have corrected this in the rephrasing (Page 5, Line 68)

5. Line 64: what is the coverage of this dataset? For those not aware of the health system in Dubai how many people go to the public vs private healthcare?

**Response:** We thank the reviewer for this comment. The dataset presented captures the patients seen in the public healthcare sector in Dubai during the study period. Based on publicly available data for 2019-2021 in the Dubai Annual Health Statistics (<https://www.dha.gov.ae/en/open-data>). The total number of outpatients seen in Dubai was 11,858,999 (2021), 8,730,496 (2020) and 11,653,351 (2019). Of these, 27.7% (2021), 33.2% (2020) and 20.5% (2019) were seen in the public healthcare sector. For inpatients, there were 402,666 in 2021 with 320,220 (2020) and 332,393 in 2019. Of these, 22%, 27.9% and 21% were seen in the public healthcare sector in 2021, 2020 and 2019 respectively.

We have now incorporated this information into the Methods section (Page 6, Lines 87-94)

**Methods**
1. Line 80: how did you define bacterial infection? This can be difficult (is it infection or just colonisation?) did you use a set code? Was it just bloodstream infections? Was it different for different patient groups / bacteria?

**Response:** The data included patients with a diagnosed/clinically suspected infection, whose samples were sent to diagnostic lab for strains identification and drug susceptibility testing as part of their clinical care. The data covered all infections and included all groups of patients. We have now mentioned this (Page 6, Lines 96-99)

2. Line 83: what types of tests are done? MIC / EUCAST etc? has it changed over time?

**Response:** The diagnostic laboratories follow the CLSI guidelines and comply with any annual updates. The EUCAST guidelines apply for colistin MIC. This has now been described in the manuscript (Page 7, Lines 102-108)

3. Line 89: which strains were sent for molecular typing?

**Response:** The list of organisms is provided in the summary data in Supplemental data on GitHub. We have now mentioned this (Page 7, Line 119-120)

4. Line 95: which patients were tested for COVID-19? Only those with symptoms?

**Response:** The datasets included available COVID-19 test results for the patient with a diagnosed infection. Patients may or may not have the symptoms at the time of test. We have now mentioned this (page 7, Lines 122-123).

5. Line 104: did you only get antibiotic prescribing data for those with an infection or all patients? Was this actual prescribing data to individual patients?

**Response:** Our study included data for patients with an infection. The data was prescription notes for the individual patient. This is already mentioned in the Methods (Page 8, Line 142)

6. Line 114: linked to above, was it all prescribing or just prescribing in those with infection?

**Response:** We obtained the prescription data for patients with a confirmed infection (Page 8, Line 142)

7. Line 118: why are you looking at seasonal trends? There should be something in the introduction on the importance of seasonality to antibiotic usage as well as the previous work on lag times between use and resistance

**Response:** Antimicrobial prescriptions are known to vary with seasons. Multiple factors may lead to changes in prescription rates at different times of the year, including differences in the prevalence of viral, bacterial, and secondary infections across seasons, difficulties in diagnosis due to similar symptoms, and shifts in travel patterns—particularly notable for Dubai as a popular tourist destination [3-5]. We, therefore, dissected the impact of the COVID-19 pandemic on this pattern. This is now included in the introduction section of our study (Page 5, Lines 69-71)

8. Line 127: should it be SARS-CoV-2 infection? COVID-19 is the disease. Similarly SARS-CoV-2 positive not COVID-19 positive? 
Line 131: tense error

**Response:** We have now fixed these (Pages 8 and 9).

9. Line 150: I can’t asses this machine learning but it’s a nice idea to look at the free text in this structure way
**Response:** We have now provided the code for the text cleansing and natural language processing (NLP) in the GitHub directory for the project.

10. Line 171: Did you look at whether the number of tests varied over time? It may be that this could drive resistance prevalence changes

**Response:** There is variation in the total number of tests, resulting from from COVID-19 related restrictions and alterations in clinical management protocols. However, since we divided the positive tests by the total number of tests, we have already factored in this variability. We have now included this in the Methods section (Page 11, Lines 201).

11. Line 184: notation not totally clear here

**Response:** We have now rewritten the section in a clearer way (page 11-12; Lines 211-221).

12. Line 193: something need in the introduction about what / why look differently at inpatient and outpatient

**Response:** We have now provided a brief explanation of why we treated the groups differently. The groups may exhibit distinct patterns of prescription rates and prescription patterns, due to the difference in disease severity [6]. We, therefore, examined the dynamics in each group separately (Page 5, Lines 71-74).

13. Line 195: was the top most frequent tested consistent over time?

**Response:** We considered only aggregated data for the antimicrobials for which full temporal data is available.

14. Line 208: shouldn’t it have been done by the type of infection? i.e. those with higher mortality rates should be separate from those with lower rates?

**Response:** We did not have the exact information on comorbidities and type of infection. However, we have now included the medical terms extracted from diagnostic notes in the logistic regression and competing risk survival model and accounted for the presence of the diagnostic terms. The diagnostic terms contain information on the type of infection, comorbidities, and other clinical features of the infection (Pages 12 and 13).

15. Line 210: did you account for any of the confounding due to longer hospital stays?

**Response:** We have now included hospitalization length and frequency in the model (Pages 12 and 13).

16. Line 219: I think this equation really needs to account for the type of infection

**Response:** We did not have the exact information about the type of infection. However, we have now included the diagnostic terms in the equation. The diagnostic terms include infection type, e.g. sepsis, comorbidities and severity of symptoms (Page 13, Line 258).

16. Line 220: there is no coefficient beta in the equation

**Response:** We have now fixed this (Page 13).

17. Line 230: there are no analysis codes in here only output data csvs – please add the code or modify this statement

**Response:** We have now included codes in the GitHub directory of the project.

**Results**

1. Line 234: what is a “confirmed” infection as opposed to the bacterial infections you talk to in the methods?

**Response:** We refer to the same concept. We have rephrased the sentence to remove this confusion (Page 15, Lines 287-291).

2. Line 234: is there any uncertainty around these values? It must have changed over time?

**Response:** We deemed the sentence ambiguous and now rephrased it to report the prescription rate and the range across years (Page 15, Lines 291-295)

3. Line 235: what is a “prescription”? is it always the same amount? E.g. 3 days of gentamicin is the same as 7 days?

**Response:** Because the data was incomplete on the drugs concentration, we calculated only the prescription count and did not consider the total drug dosage, as derived from concentrations.

4. Line 238: did you expect this sharing? Something to mention in the intro / discussion

**Response:** We have now added one sentence in the results (Page 15, Lines 297-302).

5. Line 239: how do you define dominant?

**Response:** We rephrased this to the most frequently prescribed (Page 15, Lines 297-302).

6. Line 241: checking here: this is all outpatients or just those with infections?

**Response:** Only patients with infections.

7. Line 385: Did you check all associations for confounding factors like length of hospital stay? See here for some discussion [https://urldefense.com/v3/__https://www.ncbi.nlm.nih.gov/pmc/articles/PMC8210247/__;!!Nmw4Hv0!yMIHcUH_cICsrcFo43Z88npx6MCtB7qbUhzjftxaWJxgUQUTetc7fygd8Q43LI4m0Q9_OauQqaC9xbvXIl1ndMXlEbPwBQ$](https://urldefense.com/v3/__https:/www.ncbi.nlm.nih.gov/pmc/articles/PMC8210247/__;!!Nmw4Hv0!yMIHcUH_cICsrcFo43Z88npx6MCtB7qbUhzjftxaWJxgUQUTetc7fygd8Q43LI4m0Q9_OauQqaC9xbvXIl1ndMXlEbPwBQ$)

**Response:** We have now included two key hospitalization features, i.e. hospitalization length and frequency in the model and updated the results (page 12 and 13).

8. Line 451: does the data support the MDR strains? Did you look at antibiograms? I.e. strain profiles not just individual antibiotic resistance prevalences?

**Response:** We do not have such data, as the tests were not done simultaneously. We have now rephrased the sentence (Page 24-25, Lines 517-520).

**Discussion**

1. Line 471: can you add some references here to previous work on seasonal shifts?

**Response:** We have now added the reference (Page 26, Lines 540-543).

2. Line 495: I am concerned that these mortality odds have not taken into account all the variability in sampling / length of stay and other comorbidities.

**Response:** We have now improved the model by adding hospitalization length and frequency of hospitalization.

3. Line 506: I agree that it is relatively uncommon but has been performed by others e.g. 
Especially in the UK e.g. 
[https://urldefense.com/v3/__https://www.ncbi.nlm.nih.gov/pmc/articles/PMC9303046/__;!!Nmw4Hv0!yMIHcUH_cICsrcFo43Z88npx6MCtB7qbUhzjftxaWJxgUQUTetc7fygd8Q43LI4m0Q9_OauQqaC9xbvXIl1ndMWvoAkmZg$](https://urldefense.com/v3/__https:/www.ncbi.nlm.nih.gov/pmc/articles/PMC9303046/__;!!Nmw4Hv0!yMIHcUH_cICsrcFo43Z88npx6MCtB7qbUhzjftxaWJxgUQUTetc7fygd8Q43LI4m0Q9_OauQqaC9xbvXIl1ndMWvoAkmZg$) 
[https://urldefense.com/v3/__https://www.microbiologyresearch.org/content/journal/jmm/10.1099/jmm.0.001724__;!!Nmw4Hv0!yMIHcUH_cICsrcFo43Z88npx6MCtB7qbUhzjftxaWJxgUQUTetc7fygd8Q43LI4m0Q9_OauQqaC9xbvXIl1ndMX5W5m9Lg$](https://urldefense.com/v3/__https:/www.microbiologyresearch.org/content/journal/jmm/10.1099/jmm.0.001724__;!!Nmw4Hv0!yMIHcUH_cICsrcFo43Z88npx6MCtB7qbUhzjftxaWJxgUQUTetc7fygd8Q43LI4m0Q9_OauQqaC9xbvXIl1ndMX5W5m9Lg$) 
[https://urldefense.com/v3/__https://pubmed.ncbi.nlm.nih.gov/28333200/__;!!Nmw4Hv0!yMIHcUH_cICsrcFo43Z88npx6MCtB7qbUhzjftxaWJxgUQUTetc7fygd8Q43LI4m0Q9_OauQqaC9xbvXIl1ndMWf6Euzzw$](https://urldefense.com/v3/__https:/pubmed.ncbi.nlm.nih.gov/28333200/__;!!Nmw4Hv0!yMIHcUH_cICsrcFo43Z88npx6MCtB7qbUhzjftxaWJxgUQUTetc7fygd8Q43LI4m0Q9_OauQqaC9xbvXIl1ndMWf6Euzzw$)

**Response:** We have now cited these studies and amended the statement (Page 27, Lines 575-577).

4. line 528: how many patients go to private hospitals?

**Response:** We have now indicated the percentages of patients seen in the public healthcare sector in Dubai in the methods section. The other patients are seen in the private care sector (Page 6, Lines 87-94)

5. Linked to line 418: how does these trends linking impact of COVID-19 on AMR link to the time lags you saw in the use-resistance time series analysis?

**Response:** We lacked complete records for the drugs within this cluster. In certain cases, such as chloroquine, susceptibility data were unavailable. For the drugs with available records, we showed the link. For example, we demonstrated that for macrolides like azithromycin, the consumption of which surged significantly after the onset of the pandemic, there is a correlation with elevated erythromycin resistance levels (Erythromycin is categorized in cluster 2 in Figure 7).

6. How general do you think the results are to Dubai? Can you make any broader conclusions?

**Response:** Our results align qualitatively with the trends reported in other countries, as we have discussed consistently throughout the manuscript. For instance, our findings concerning antimicrobial consumption and seasonal patterns are in line with prior reports. We have also observed a consistent increase in the prescription of certain antimicrobials, such as hydroxychloroquine. Due to the extensive and comprehensive nature of our dataset, we believe that the insights gleaned can be applied to other settings to some extent. However, detailed electronic health record (EHR) data on antimicrobial consumption and resistance is still growing. To further explore the commonalities and distinct aspects, future comparative studies with other regions is warranted. We have now mentioned this in the limitation section (Page 28-29, Lines 605-611).

7. Figure 2: Can you explain why you highlight blue / red in the top? COVID linked to the bottom graph right?

**Response:** Blue and red designates terms related to COVID infection, i.e. <<COVID>> and <<coronavirus>>. We have now mentioned this in the caption.

8. What does tf-df stand for?

**Response:** Tf-idf stands for Term Frequency - Inverse Document Frequency. We have corrected and mentioned this.

9. Only inpatient use is shown in the top correct?

**Response:** Yes as for outpatient COVID was not found as a top important term

10. Figure 3:
Blue line “covid start”: can you be more specific? Was this lockdowns in Dubai? And it is “COVID-19”

**Response:** It corresponds to the time that the first case was identified. We have now detailed this (Line 672).

11. Figure 4: 
-       Covid should be capitalised
-       Is “covid onset” the same as covid start in Figure 3? Please be consistent. And different to “COVID-19 start” in Figure 5

**Response:** We have now fixed this

12. (c) and (d) legend explanation is missing something? 
**Response:** We have now fixed these.

13. Figure 5: 
A: why does resistance rise before rising consumption levels?

**Response:** Resistance increases before the consumption level, however the extent of the increase was not big enough to be visually noticeable. The two plots describe two different features with different dynamics. We demonstrated the link between consumption and resistance but there are factors determining the dynamics.

14. Line 640: “for” the resistance level?

**Response:** This has been addressed.

15. Figure 6:
Why show both the estimate and the odds ratio? How do they relate? Are they both accounting for ethnicity age bmi and gender? 
**Response:** Odds ratio test does not account for the confounding factors. The estimate from logistic regression and competing regression analysis accounts for this. We have now detailed this in the caption.

16. Figure 7: Is A trends or clusters? B is trends?

**Response:** We have now corrected this.


--------------------

1.	Austin, P.C. and J.P. Fine, *Practical recommendations for reporting Fine-Gray model analyses for competing risk data.* Stat Med, 2017. **36**(27): p. 4391-4400.

2.	Dignam, J.J., Q. Zhang, and M. Kocherginsky, *The use and interpretation of competing risks regression models.* Clin Cancer Res, 2012. **18**(8): p. 2301-8.

3.	Butler, C.C., et al., *Variation in antibiotic prescribing and its impact on recovery in patients with acute cough in primary care: prospective study in 13 countries.* BMJ, 2009. **338**: p. b2242.

4.	Tedijanto, C., et al., *Drivers of Seasonal Variation in Tuberculosis Incidence: Insights from a Systematic Review and Mathematical Model.* Epidemiology, 2018. **29**(6): p. 857-866.

5.	Suda, K.J., et al., *Trends and seasonal variation in outpatient antibiotic prescription rates in the United States, 2006 to 2010.* Antimicrob Agents Chemother, 2014. **58**(5): p. 2763-6.

6.	Yin, X., et al., *A systematic review of antibiotic utilization in China.* J Antimicrob Chemother, 2013. **68**(11): p. 2445-52.
